# A phenolic amide (LyA) isolated from the fruits of *Lycium barbarum* protects against cerebral ischemia–reperfusion injury via PKCε/Nrf2/HO-1 pathway

**DOI:** 10.18632/aging.102578

**Published:** 2019-12-26

**Authors:** Kai Gao, Meiyou Liu, Yi Ding, Minna Yao, Yanrong Zhu, Jinyi Zhao, Lianghua Cheng, Juan Bai, Fan Wang, Jinyi Cao, Jiankang Li, Haifeng Tang, Yanyan Jia, Aidong Wen

**Affiliations:** 1 Department of Pharmacy, Xijing Hospital, Fourth Military Medical University, Xi’an 710032, China; 2 Institute of Materia Medica, School of Pharmacy, Fourth Military Medical University, Xi’an 710032, China

**Keywords:** ischemic stroke, Lyciumamide A, oxidative stress, PKCε, Nrf2, HO-1

## Abstract

Lyciumamide A (LyA), a dimer of phenolic amide isolated from the fruits of *Lycium barbarum,* has been confirmed to possess potent antioxidant activity. This study was aimed to investigate the neuroprotection and molecular mechanisms of LyA against cerebral ischemia/reperfusion (I/R) injury via improving antioxidant activity. The model of middle cerebral artery occlusion (MCAO) and SH-SY5Y cells induced by oxygen and glucose deprivation (OGD) were adopted to verify the neuroprotective effects and the potential pharmacology mechanisms of LyA *in vivo* and *in vitro*. In MCAO model, treatment with LyA significantly improved neurologic score, reduced infarct volume, and relieved oxidative stress injury at 48 h after reperfusion. Meanwhile, LyA markedly increased the transcription Nrf2 and HO-1 expressions in the ischemic cerebral cortex. In vitro results showed that LyA protected differentiated SH-SY5Y cells against OGD-induced injury. LyA significantly decreased the expression of caspase-3 and the Bax/Bcl-2 ratio. But knockdown of Nrf2 or HO-1 attenuated the protective effect of LyA. Similarly, knockdown of protein kinase Cε (PKCε) inhibited LyA-induced Nrf2/HO-1 activation, and abated its protective effects. In conclusion, this study firstly demonstrated that LyA protects against cerebral I/R injury, ameliorates oxidative damage and neuronal apoptosis, partly via activation of PKCε/Nrf2/HO-1 pathway.

## INTRODUCTION

Stroke is the second leading cause of death in the world and is a major cause of adult disability [1]. After stroke, during the cerebral ischemia/reperfusion injury (I/R), excessive levels of reactive oxygen species (ROS) can due to an imbalance between oxidants and antioxidants leading to oxidative stress and play a vital role [2, 3]. Now, antioxidants from natural medicine have been considered for the prevention and treatment of ischemic stroke.

Previously, our lab had conducted a number of studies on the neuroprotective effect of natural antioxidants through the antioxidant pathway Nrf2/HO-1 [4–7]. Nuclear factor erythroid 2-related factor 2 (Nrf2) plays an important role in protecting cells from oxidative stress. It has been reported that heme oxygenase-1 (HO-1) has the most antioxidant-responsive elements (AREs) on its promoter, making it a highly effective therapeutic target for preventing brain damage after ischemic stroke [8–10]. Protein kinase C (PKC) is a family of protein kinase enzymes composed of 10 serine/threonine kinases [11]. It has been shown that the activation of PKC mediates the stimulation of Nrf2 in response to oxidative stress [12]. These findings have motivated us to explore natural Chinese herbal compounds with potential antioxidant effects to prevent cerebral ischemic stroke.

**Figure 1:**
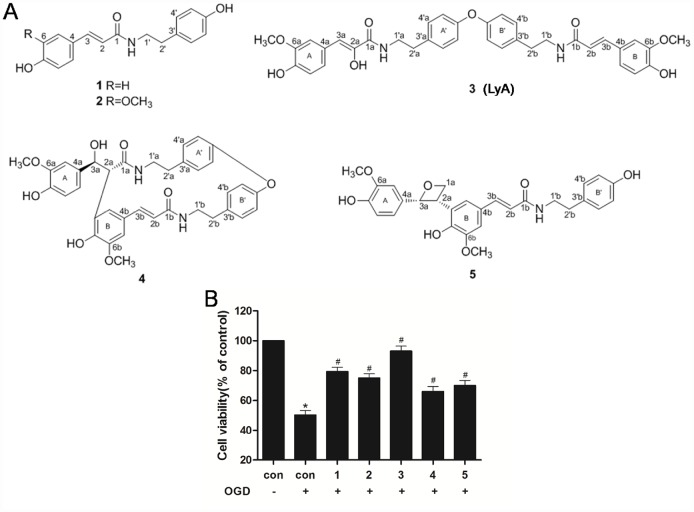
**Effects of phenolic amides and OGD on cell viability.** (**A**) Chemical structure of phenolic amides (1-5). Thereinto, the compound 3 is Lyciumamide A (LyA). (**B**) MTT assay was employed to investigate the protective effects of phenolic amides against OGD-induced cytotoxicity. The concentrations of these compounds is 40 μM. Data were represented as means ± SD (n=6). ^*^ *p* < 0.05 compared with control group; ^#^ *p* < 0.05 compared with OGD group.

Lyciumamide A (LyA), a new dimer of phenolic amide isolated from the fruits of *Lycium barbarum,* is a powerful natural antioxidant. The fruits of *Lycium barbarum* (family Solanaceae), a traditional Chinese herb used in many countries as traditional medicine and nutritional food, has significant antioxidative and immunomodulatory properties in the context of anti-tumor, neuroprotection and age-related diseases including atherosclerosis, neurodegeneration and diabetes [13, 14]. However, the research on the antioxidant constituents of the fruits mainly focused on the effective parts. Olatunji et al reported that the ethyl acetate extract of wolfberry exerted a neuroprotective effect through its antioxidant capacity, but the specific active ingredient of ethyl acetate extract was not clear [15]. Previously, our research firstly discovered a range of antioxidants (phenolic amides including dimer structure) by the help of activity guided chromatography for antioxidants in extract layer from ethyl acetate of the fruits [16]. Thereinto, we speculated that LyA exerted the strongest neuroprotective effect, and its underlying mechanism has not been studied (Figure 1).

In the current study, the model of middle cerebral artery occlusion (MCAO) and SH-SY5Y cells induced by oxygen and glucose deprivation (OGD) were adopted to verify the neuroprotective effects of LyA and the potential mechanisms *in vivo* and *in vitro*. The aim of this study is to investigate whether LyA prevents cerebral I/R injury via PKCε/Nrf2/HO-1 pathway.

## RESULTS

### Phenolic amides protected SH-SY5Y cells against OGD on cell viability

MTT assay was employed to assess the cell viability of SH-SY5Y cells induced by OGD in the experiment. It was found that OGD-induced a significant decrease in cell viability as compared to control cells (Figure 1B). Meanwhile, treatment with phenolic amides (1-5) (40 μM) could significantly improve cell viability (*p* < 0.05), of which compound 3 (LyA) has the strongest activity (Figure 1B).

### LyA protected against cerebral I/R injury

**Figure 2:**
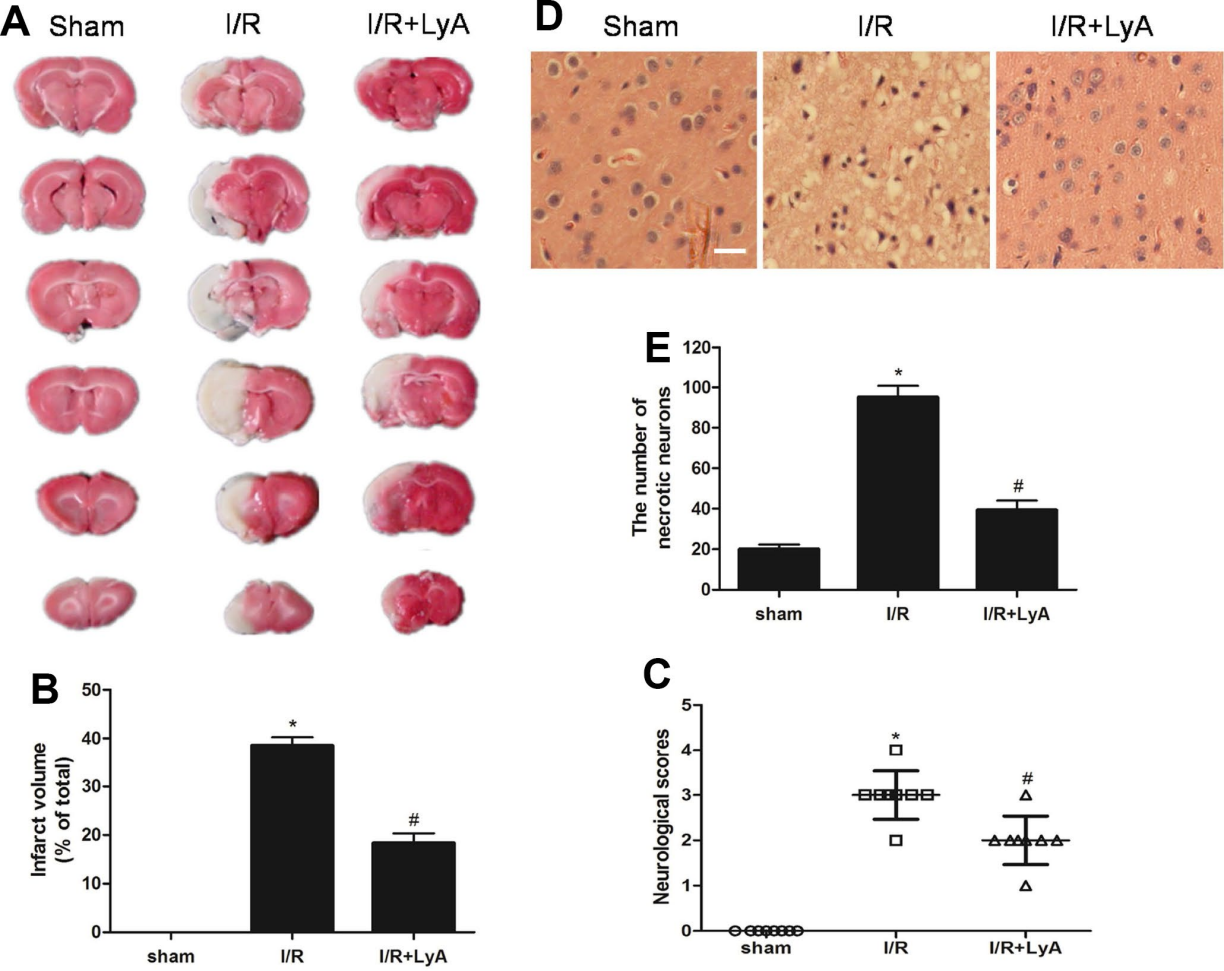
**LyA protects against cerebral ischemic-reperfusion injury.** (**A**) TTC staining of the cerebral infarct in the sham, control and treatment with LyA groups. (**B**) The columnar diagram for the infarct volume of brains in each group (n=6). (**C**) Neurological scores of rats at 48 h after cerebral I/R for each group (n=8). (**D**) H-E stains of coronal sections from the ischemic cerebral cortex (*scale bar* 100 μm). (**E**) Necrotic neurons were counted and analyzed in each group (n=6). All data, except for neurological scores, were expressed as mean ± SD. ^*^ *p* < 0.05 compared with sham group; ^#^ *p* < 0.05 compared with I/R group.

The neuroprotective effect of LyA against I/R injury by MCAO was evaluated through infarct volume. As shown in Figure 2A and 2B, MCAO resulted in a large infarct volume in the brain. Meanwhile, LyA (40 mg/kg) could significantly reduce the infarct volume compared with I/R group (*p* < 0.05). To evaluate the neurological function, neurological deficit grading system was carried out. In agreement with infarct volume measurement, LyA treatment significantly reduced the neurological deficit score compared with I/R group (*p* < 0.05, Figure 2C). Furthermore, the protective effect of LyA on cerebral I/R injury was confirmed by histological observation. Results as shown in Figure 2D and 2E, the cells of cortex in sham rats showed an orderly arrangement, the cell outline was clear, the structure was compact, and the nucleolus was clearly visible. In I/R group, the number of cells was decreased and the cells were arranged irregularly in ischemic peri-infarct of cerebral cortex. Most of them were shrunken with a triangulated pycnotic nucleus. In contrast, neuronal damage was substantially reduced in the LyA + I/R group (*p* < 0.05 vs. I/R group).

### LyA attenuated oxidative stress

SOD and GPx activity in the cortex of the I/R group was decreased compared with the sham group (*p* < 0.05, Table 1), while LyA markedly restored them (*p* < 0.05, Table 1). The MDA level in the cortex of the I/R group was evidently increased compared with the sham group (*p* < 0.05, Table 1). And a significant decrease in MDA levels was observed in the LyA + I/R group compared to the I/R group (*p* < 0.05, Table 1).

**Table 1:** Levels of SOD, GPx, and MDA in the cortex at 48 h after reperfusion in each group.

	**SOD, U/mg**	**GPx, U/mg**	**MDA, nmol/mg**
Sham	156.0 ±9.3	72.1±6.8	7.6±1.0
I/R	80.5±6.1^*^	27.6±3.6^*^	24.8±3.4^*^
LyA+I/R	145.3±8.0^#^	64.2±6.3^#^	11. 8±1.1^#^

Data were represented as means ± SD (n=6 animals for each group).

^·^ *p*<0.05 I/R group versus sham group; ^#^ *p*<0.05 LyA + I/R group versus I/R group.

### LyA promoted the expression of Nrf2 and HO-1

**Figure 3:**
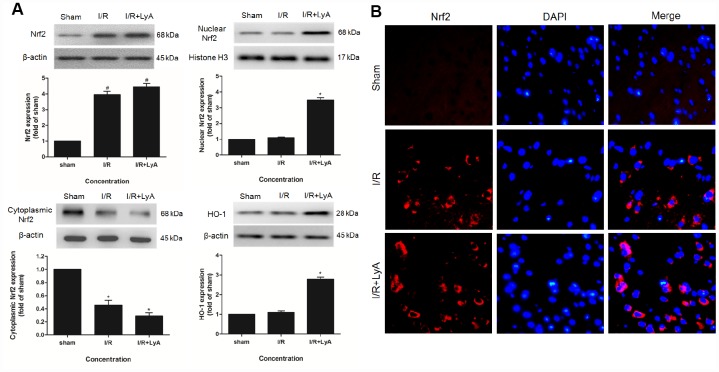
**LyA Promoted the Expression of Nrf2 and HO-1.** (**A**) Protein expressions of Nuclear Nrf2, Cytoplasmic Nrf2 and HO-1 were evaluated by Western blot analysis. (**B**) The immunofluorescence staining of Nrf2 with DAPI (400 x). Data were presented as mean ± SD (n = 6). ^*^ *p* < 0.05 compared with I/R group.

To determine whether Nrf2 or/and HO-1 are involved in the neuroprotective effect of LyA, we analyzed ischemic brain tissue by western blot. As shown in Figure 3A, compared with the sham group, I/R group and LyA + I/R group increased the expression of total Nrf2 protein, but LyA does not significantly increase the expression of total Nrf2 compared to the I/R group. Treatment with LyA obviously increased the protein expression of nuclear Nrf2 and cytoplasmic HO-1 (Figure 3A) (*p* < 0.05). And Nrf2 was accumulated in the nucleus while cytoplasmic Nrf2 levels were decreased (Figure 3A).

Consistently, immunofluorescence staining also showed that the expression of Nrf2 in the cortex was upregulated by LyA after ischemia (Figure 3B). In sham group, few cells were stained by Nrf2. In the I/R group and LyA + I/R group, the number of cells stained by Nrf2 significantly increased. And LyA promoted the transportation of Nrf2 from the cytoplasm to the nucleus compared with I/R group, which indicated that the Nrf2 pathway may have a critical role in the LyA mediated neuroprotection against I/R injuries in rats.

### LyA protected SH-SY5Y cells against OGD-induced injury

**Figure 4:**
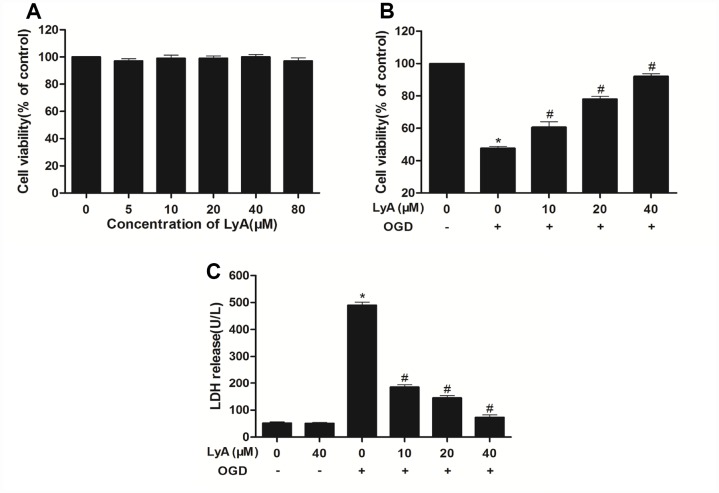
**Effects of LyA and OGD on cell viability.** (**A**) Cells were incubated in different concentrations of LyA alone. (**B**) MTT assay was employed to investigate the protective effect of LyA against OGD-induced cytotoxicity. (**C**) Cell death was further confirmed by measuring LDH leakage. Data were represented as means ± SD (n=6). ^*^ *p* < 0.05 compared with control group; ^#^ *p* < 0.05 compared with OGD group.

Similarly, MTT assay was employed to explore the effect of LyA against OGD-induced injury. It was found that OGD-induced a significant decrease in cell viability as compared to control cells (Figure 4B). In Figure 4A, different concentrations of LyA alone cannot affect the cell viability. Treatment with LyA (10, 20, 40 μM) showed an effective neuroprotection effect on OGD-induced neuronal damage, and the cell viability was obviously increased (Figure 4B).

To further investigate the protective effect of LyA against OGD-induced cytotoxicity, the LDH leakage was measured. As shown in Figure 4C, the LDH release in OGD group was much more than the control group (*p* < 0.05). Conversely, LyA (10, 20, 40 μΜ) treatment lowered the LDH release in a dose-dependent manner. LyA alone had no effect on LDH levels.

### LyA attenuated OGD-induced apoptosis in SH-SY5Y cells

**Figure 5:**
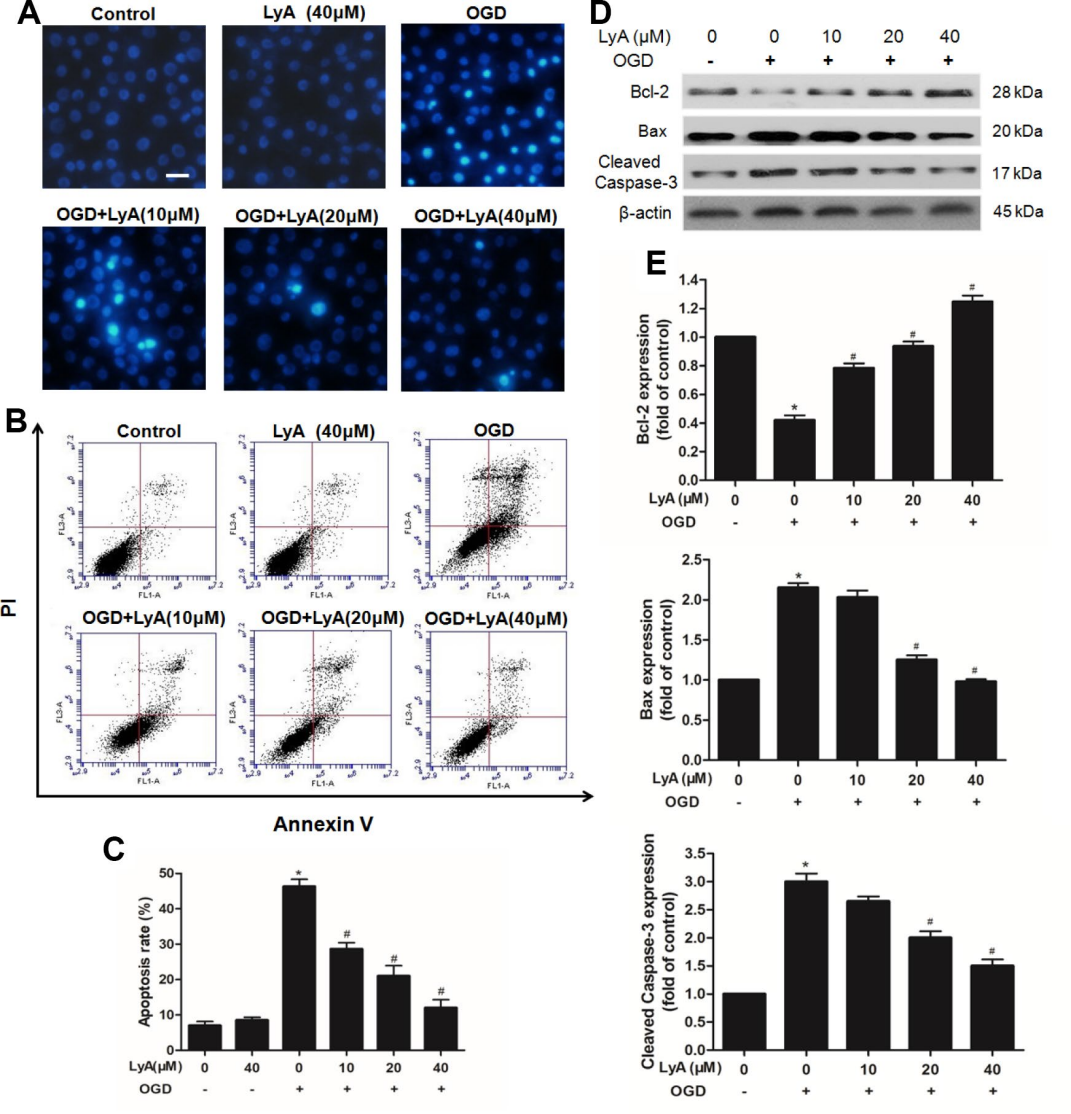
**Effects of LyA on apoptotic in SH-SY5Y cells.** (**A**) Hoechst 33342 staining (*scale bar* 100 μm). (**B**) Effects of LyA on OGD-induced apoptosis after AV-PI double stain. (**C**) The quantitative analysis of apoptotic cells. (**D**) Protein expressions of bax, bcl-2 and cleaved caspase-3 were evaluated by western blot analysis. (**E**) Relative expressions are calculated and are shown here. Data were represented as means ± SD (n=6). ^*^ *p* < 0.05 compared with control group; ^#^ *p* < 0.05 compared with OGD group.

As shown in Figure 5A, after OGD, the SH-SY5Y cells showed that the apoptotic cell nucleus was converted into light blue fluorescence contrasted to viable cell with faint blue fluorescence. However, treatment with different concentration of LyA (10, 20, 40 μΜ) reduced the apoptotic cells in a concentration-dependent manner.

AnnexinV-PI double staining assay was used to further distinguish the characteristics of apoptotic and necrotic cells induced by OGD with or without LyA. As shown in Figure 5B and 5C, treatment with OGD increased the percentage of apoptotic cells compared to the control group (*p* < 0.05). Pretreatment with LyA (10, 20, 40 μΜ) before treatment with OGD could distinctly reduce the percentage of apoptotic cells in a concentration- dependent manner (*p* < 0.05). In addition, LyA alone did not display any obvious effect.

Consistently, western blot also showed that OGD increased the expression of Bax and cleaved Caspase-3, and reduced the expression of Bcl-2, LyA treatment reversed this trend in a dose-dependent manner (Figure 5D and 5E).

### Effects of LyA treatment on PKCε activation and Nrf2/HO-1 signaling pathway in cells

**Figure 6:**
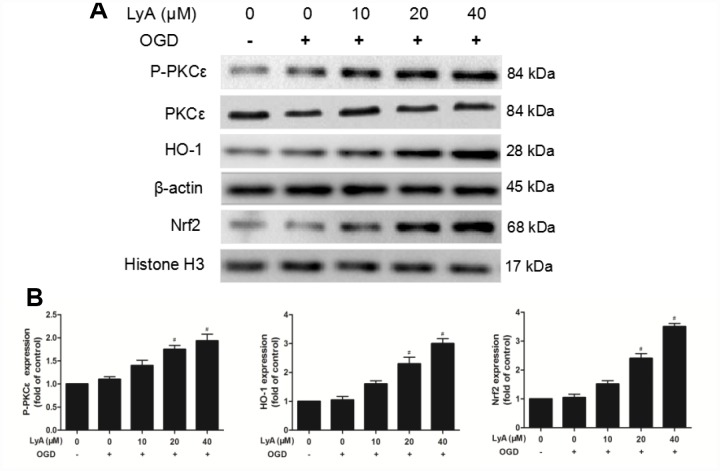
**Effect of LyA treatment on PKCε phosphorylation, activation of Nrf2/HO-1 pathway in SH-SY5Y cells.** (**A**) Representative bands of each protein are presented. (**B**) Relative expressions are calculated and are shown here. Data were represented as means ± SD (n=6). ^*^ *p* < 0.05 compared with control group; ^#^ *p* < 0.05 compared with OGD group.

To investigate the effects of LyA on the expression of p-PKCε, Nrf2 and HO-1 in differentiated SH-SY5Y cells, we analyzed the cells by western blot. As shown in Figure 6A and 6B, the differentiated cells incubated with OGD slightly increased the protein expressions of p-PKCε, nuclear Nrf2 and cytoplasmic HO-1, but there was no statistical difference between the control group and the OGD group. Interestingly, LyA markedly increased the expression levels of p-PKCε, Nrf2 and HO-1 in a dose-dependent manner compared to the OGD group.

### The neuroprotection of LyA involved the Nrf2/HO-1 pathway

**Figure 7:**
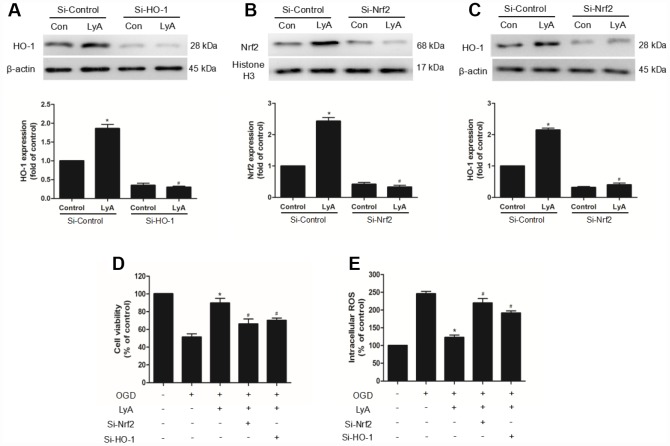
**The neuroprotection of LyA involved the Nrf2/HO-1 pathway.** (**A**–**C**) Cells were transfected with control or HO-1 and Nrf2 siRNA for 48 h, followed by treatment with 40 μM LyA for 8 h. HO-1 and Nrf2 expression levels were analyzed by western blotting. Data were presented as mean ± SD (n =6). ^*^ *p* < 0.05 vs si-control group without LyA; ^#^ *p* < 0.05 vs si-control group with LyA. (**D**) Cells were treated for 48 h with control or siRNA, and then treated with 40 μM LyA for 8 h before being subjected to 60 min OGD followed at 24 h by the MTT assay. (**E**) Intracellular ROS level. Data were represented as means ± SD (n=6). ^*^ *p* < 0.05 compared with OGD group; ^#^ *p* < 0.05 compared with OGD + LyA group.

To confirm the role of Nrf2/HO-1 pathway in LyA-mediated neuroprotection, we transfected differentiated SH-SY5Y cells with control (si-control), HO-1- specific (si-HO-1), or Nrf2-specific (si-Nrf2) siRNA for 48h. The efficiency of RNA interference was evaluated by western blot. As shown in Figure 7, HO-1 siRNA obviously decreased the expression of HO-1 under vehicle or LyA treatment, but control siRNA didn’t have such effect (Figure 7A). Similar to the HO-1 knockdown results, Nrf2 knockdown significantly reduced Nrf2 nuclear translocation and HO-1 expression (Figure 7B and 7C). In cell survival experiment, knockdown of Nrf2 or HO-1 inhibited increased cell viability of LyA under OGD (Figure 7D). Consistently, knockdown of Nrf2 or HO-1 increased ROS level compared to the si-control group, respectively (Figure 7E). Taken together, LyA effectively exerted anti-oxidative damage by activating Nrf2/HO-1 pathway.

### PKCε mediated LyA-induced neuroprotection, Nrf2 nuclear translocation, and HO-1 up-regulation

**Figure 8:**
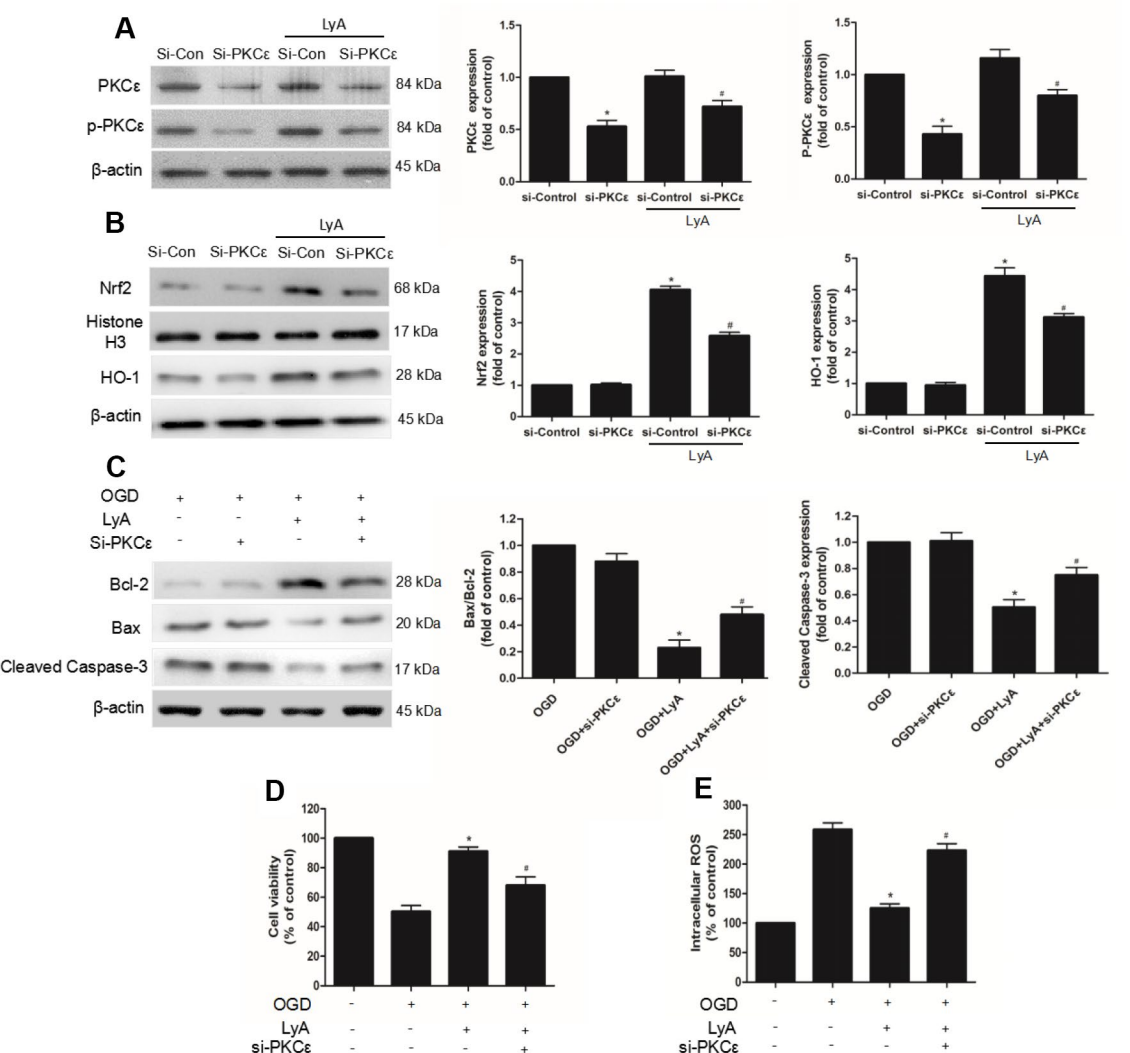
**PKCε mediated LyA-induced neuroprotection, Nrf2 nuclear translocation, and HO-1 upregulation.** (**A**–**C**) Cells were transfected with control or PKCε siRNA for 48 h, followed by treatment with 40 μM LyA for 8 h. p-PKCε, PKCε, HO-1, Nrf2, bax, bcl-2 and cleaved caspase-3 expression levels were analyzed by western blotting. Data were presented as mean ± SD (n =6). ^*^ *p* < 0.05 vs si-control group without LyA; ^#^ *p* < 0.05 vs si-control group with LyA. (**D**) Cells were treated for 48 h with control or siRNA, and then treated with 40 μM LyA for 8 h before being subjected to 60 min OGD followed at 24 h by the MTT assay. (**E**) Intracellular ROS level. Data were represented as means ± SD (n=6). ^*^ *p* < 0.05 compared with OGD group; ^#^ *p* < 0.05 compared with OGD + LyA group.

To investigate the role of PKCε in LyA-induced neuroprotection, siRNA was used to knockdown PKCε. The efficiency of RNA interference was evaluated by western blot. As shown in Figure 8, PKCε siRNA obviously decreased the expression of p-PKCε and PKCε under vehicle or LyA treatment, but control siRNA didn’t have such effect (Figure 8A). Moreover, in siRNA group LyA failed to induce nuclear translocation of Nrf2, upregulation of HO-1, and whereas control siRNA had no such effect (Figure 8B and 8C). The Bax/Bcl2 ratio and the expression of cleaved Caspase-3 were down-regulated by LyA pretreatment, which was reversed by PKCε-targeted siRNA (Figure 8D). Similar in cell survival experiment, knockdown of PKCε inhibited increased cell viability of LyA under OGD (Figure 8E). Consistently, knockdown of PKCε increased ROS level compared to the si-control group, respectively (Figure 8F). The above results suggested that LyA-induced neuroprotection was highly dependent on an increase in PKCε expression, possibly through its effect on activation of antioxidant pathways such as Nrf2/HO-1.

## DISCUSSION

In this study, we demonstrated that LyA has protective effect against cerebral I/R injury in a MCAO model and OGD-stimulated SH-SY5Ycells. Specifically, LyA improved neurologic score, reduced infarct volume ratio and the number of necrotic neuron. In addition, the influence of LyA on cerebral antioxidant enzymes after cerebral ischemia and reperfusion was explored. Two enzymes, superoxide dismutase (SOD) and glutathione peroxidase (GPx), play a key role in I/R injury [17]. Our results showed that LyA decreased reactive oxygen species (ROS), malondialdehyde (MDA) production, and increased SOD and GPx activity by inhibiting oxidative stress. Numerous studies have indicated that OGD injury can induce neuronal apoptosis [18]. Therefore, the neuronal apoptosis was assessed by Hoechst 33342 staining and AnnexinV-PI double staining in this study. These results showed that pretreatment with LyA (10, 20, 40 μΜ) before treatment with OGD could distinctly reduce the percentage of apoptotic cells in a concentration-dependent manner. At the same time, our studies also clarified that the protective effect of LyA is mediated by stimulation of PKCε, activation of Nrf2 nuclear translocation and up-regulation of HO-1.

Nrf2 is an important transcription factor regulating oxidative stress and has been proved to reduce oxidative stress in stroke [19]. Activation of Nrf2 plays an important role in enhancing the upregulation of the expression of several antioxidant enzymes, including heme oxygenase (HO-1), glutathione-S-transferase (GST) and NADPH quinone oxidoreductase (NQO1), which protect the cell from the harmful effects of ROS on cerebral I/R injury [7, 8]. Among them, it is reported that HO-1 has the most antioxidant-responsive elements (AREs) on its promoter and is a highly effective therapeutic target for preventing brain damage after ischemic stroke. Our results showed that LyA markedly increased the expression of nuclear Nrf2 and cytoplasmic HO-1 in ischemic cerebral cortex at 48 h after MCAO. In accordance with the in vivo result, LyA also significantly induced Nrf2 nuclear translocation and HO-1 upregulation in vitro. To further study the Nrf2/HO-1 involved in LyA-induced neuroprotection, we used RNA interference targeting Nrf2 or HO-1. The results demonstrated that Nrf2 and HO-1 knockdown distinctly reduced the neuroprotection effect of LyA and increased intracellular ROS levels. Meanwhile, Nrf2 knockdown inhibited LyA-induced HO-1 expression. This indicated that LyA-induced upregulation of HO-1 is Nrf2-dependent.

PKC is known to play a role in the nuclear translocation of Nrf2, which responds to oxidative stress by regulating its phosphorylation reaction, resulting in the activation of AREs and subsequent production of antioxidant enzymes [12, 20]. Experimental evidence supports the association between Nrf2 activation and the PKC pathway. In vitro model of neurodegenerative disease, activation of the PKC signalling pathway regulates Nrf2. PKCε is a novel member of PKC subfamily that protects neuroblastoma SK-N-SH cells from cell death induced by high density culture [21]. Activation of PKCε increases the mitochondrial membrane potential and decreases H_2_O_2_ production in hippocampal synaptosomes, and exhibits its neuroprotective effect [22]. Moreover, Mitochondrial PKCε activation upregulates the expression of anti-apoptotic protein Bcl-2 and inhibits the expression of proapoptotic protein Bax [23]. Studies also showed that Nrf2 participated in the increased expression of anti-apoptotic factors, such as Bcl-2 and Bcl-xL, and also reduced the activity of caspase-3 [24–26]. In our vitro experiment, LyA preconditioning further significantly increased p-PKCε expression in a dose-dependent manner. Then, siRNA was used to knock down PKCε expression, and it could markedly block LyA-induced neuroprotection against OGD injury with increase of intracellular ROS levels. Similar to our results: we discovered that Nrf2/HO-1 signaling was activated, Bax and cleaved caspase-3 were down-regulated, and Bcl2 was up-regulated by LyA pretreatment, which was reversed by PKCε-targeted siRNA. These results suggest that PKCε is necessary for LyA-induced Nrf2 nuclear translocation, HO-1 up-regulation, and neuroprotection.

However, this study focused on the protective effect of LyA against cerebral I/R injury via improving antioxidant activity but did not explore in detail whether other signaling pathways contribute to the effects of LyA. In Figures 7D, 7E and 8, we expect to see significant abolishment effects of LyA when PKCε/Nrf2/HO-1 was knockdown in conjunction of LyA treatment. Yet the phenotype was rather mild, suggesting there may be other pathway mediating LyA function. We should screen other signal pathways through inhibitors or other methods. Moreover, because we only considered that the expression of Nrf2 in the nucleus can also demonstrate the effect of LyA, so the expression of phosphorylated Nrf2, cytoplasmic Nrf2 are not measured separately. We should consider the phosphorylation of Nrf2 at Ser40 and cytoplasmic Nrf2 in the PKC/Nrf2 activation. Another limitation was that, in this study, we only explored the possible mechanisms through siRNA, but didn’t test the contrary. We ignored the verification of the results from the opposite side, we should also consider the role of overexpression. Last but not least, the analysis of the mechanism is not deep enough, more NRF2-gene targets and direct oxidation indicators should be detected, and some results can be verified by more different types of experiments. Although the mechanisms are poorly defined, we believe that PKCε/Nrf2/HO-1 induced by LyA decreased ROS and that it is responsible, at least in part, for the protective effects against OGD.

In summary, our study firstly confirms that LyA, a dimer of phenolic amide isolated from the fruits of *Lycium barbarum,* exerts significant neuroprotective effects through its antioxidant capacity in vivo and in vitro. Our results show that LyA ameliorates oxidative damage and neuronal apoptosis via PKCε/Nrf2/HO-1 Pathway. it revealed that phenolic amides are the important antioxidant constituents in the ethyl acetate extract of wolfberry with neuroprotective effects. The evidence in the present study suggests that LyA may be a potential compound for the treatment of cerebral ischemia/ reperfusion injury partly via PKCε/Nrf2/HO-1 pathway.

## MATERIALS AND METHODS

### Reagents and antibodies

LyA (purity>98%) was isolated and purified as previously reported [16]. Dulbecco’s modified Eagle’s medium (DMEM) and fetal bovine serum (FBS) were purchased from Gibco (Gaithersburg, MD, USA). The dihydroethidium (DHE), superoxide dismutase (SOD), glutathione peroxidase (GPx), and malondialdehyde (MDA) assay kits, and the cytoplasmic and nuclear protein extraction kits were purchased from Nanjing Jiancheng Bioengineering Institute (Nanjing, China). The anti-p-PKCε, anti-PKCε, anti-Nrf2 were purchased from Abcam (Cambridge, MA, USA). The anti-HO-1, anti-Bcl2, anti-Bax, anti-cleaved caspase3, anti-β-actin, anti-histone H3 were purchased from Cell Signaling Technology (Boston, MA, USA). 3-(4, 5-Dimethylthiazol-2-yl)-2, 5-diphenyltetrazolium bromide (MTT), dimethylsulfoxide, Hoechst 33342 and all other chemicals were purchased from Sigma-Chemical (St. Louis, MO, USA).

### Middle cerebral artery occlusion (MCAO)

Adult male Sprague-Dawley rats weighing 280 ± 20 g were supplied by the Experimental Animal Center of the Fourth Military. The male rats were exposed to food and water available in a room under the conditions of controlled temperature of 22 ± 3 °C, humidity of 60 ± 5 % and 12 h of light/12 h of dark circulation. All experimental procedures were carried out according to protocols approved by the Animal Care and Use Committee of the Fourth Military Medical University (xjp0211). The MCAO model was performed according to previously described methods with some modifications [27]. In brief, rodents were anaesthetized (1.5% isoflurane in 70% nitrous oxide and 30% oxygen). The right common, external, internal carotid arteries (ICA) and external carotid artery (ECA) were exposed and carefully isolated. The 3-0 monofilament nylon suture (Ethicon, Inc., Osaka, Japan) was introduced from the ECA into the right ICA to occlude the origin of the right middle cerebral artery (MCA). The suture was slowly withdrawn 2 h after the induction of ischemia. Cerebral blood flow (CBF) in the ipsilateral cortex (2 mm posterior and 5 mm lateral to bregma) was monitored by laser Doppler flowmetry (Perimed AB, PeriFlux System 5000, Stockholm, Sweden). Sham operated rats operated in the same manner, but the MCA was not occluded. Animals that did not show a CBF reduction of at least 70% and animals that died after induction of saturation were excluded from the groups.

Animals were randomly divided into three groups using the random number table generated by SPSS 18.0 (SPSS Inc., Chicago, IL, USA): sham, I/R, and I/R + LyA. Sham-operated rats underwent surgery and exposed right ICA and right ECA, but did not suffer MCAO. The MCAO rats were administered the physiological saline or LyA (40 mg/kg) by peritoneal injection immediately after the surgery. In all the three groups, eight rats were used for infarct size measurement, six rats were used for hematoxylin–eosin (HE) staining and necrotic neurons, six rats were used for determination of oxidative stress, six rats were used for western blotting, and six rats were used for immunostaining. In our preliminary experiment, using different doses of LyA (20, 40 and 80 mg/kg), 40 mg/kg could significantly reduce infarct volumes and improve neurological deficit scores in rats, but there was no difference between 40 mg/kg and 80 mg/kg.

### Measurement of neurological deficit score and infarct size

Neurological deficits in each group (n=8) of animals were determined after reperfusion 48 h according to Longa's five score system [27]. Ischemic lesion volume was assessed via triphenyl tetrazolium chloride (TTC) staining. Briefly, after reperfusion, the rats were sacrificed by decapitation and the brain was kept at −20 °C for 30 min. Coronal sections (2 mm) of the frozen brains were cut and stained with 1% TTC for 30 min at 37 °C, and then transferred to 4 % formalin buffer solution for 24 h. Brain slices were photographed using a digital camera and the infarct volume of each slice was calculated by image analysis system software. To compensate for brain edema, the corrected infarct area = the measured infarct area × {1-[(ipsilateral hemisphere area - contralateral hemisphere area)/contralateral hemisphere]}.

### Histological observation

To assess histological damage, rats were sacrificed after 48 h of MCAO by perfusion, and then perfused with physiological saline solution at 4 °C. 4 % (v/v) paraformaldehyde was fresh prepared in 0.1 M phosphate buffered saline (PBS) buffer (pH 7.4). Brain tissue was removed and fixed with 4% (w/v) paraformaldehyde for 24 h. The brain block was then embedded in paraffin and cut into 5 mm coronal sections. The central region of the infarction and the peri-infarct cortex sections were stained with hematoxylin–eosin (HE) using standard methods. The number of necrotic neurons in the ischemic cortex was blindly counted by light microscopy at × 400 magnification (BX51; Olympus, Tokyo, Japan) in three different slides per rat. The data for each group of six rats were averaged.

### Immunofluorescence staining

In short, the paraffin sections, 5 mm thick, which were drawn 48 h after reperfusion, were first deparaffinized in xylene, rehydrated with various grades of ethanol, and placed in Tris-EDTA (pH 9.0) for antigen retrieval. The sections were first incubated with normal goat serum for 1 h at room temperature. Then were incubated at 4 °C over night with anti-Nrf2 (ab31163, Abcam). Sections were then incubated with Alexa Fluor® 594 donkey-rabbit IgG secondary antibodies for 2 h. Then were incubated with DAPI for nuclear counterstaining. Finally, the sections were coverslipped with cover glass. The stained sections were examined under a fluorescence microscope (Olympus, Tokyo, Japan).

### Assessment of oxidative damage

The cerebral cortex tissue was first homogenized in 2 ml of 10 mM phosphate buffer (pH 7.4). Then centrifugate at 12,000 g for 20 min, and evaluate the superoxide dismutase (SOD), glutathione peroxidase (GPx) and malondialdehyde (MDA) content in the supernatant by spectrophotometry using the corresponding kits (Nanjing Jiancheng Biochemistry Co., Nanjing, China). Finally, the protein concentrations were determined by the Bradford method. The levels of SOD, GPx and MDA were expressed as units / mg protein, units / mg protein and, nmol / mg protein.

### Cell culture and treatment

Human neuroblastoma SH-SY5Y cells were cultured in DMEM supplemented with 10 % (v/v) fetal bovine serum, 100 unit/ml penicillin and 100 μg/ml streptomycin (Life Technologies). For differentiation experiments, SH-SY5Y cells were cultured in a mixture containing 1:1 Ham’s F12 and DMEM supplemented with 10 % (v/v) FBS, 100 U/ml penicillin, and streptomycin. After 24 hours, 10 μM ATRA was added into the medium to stimulate the cells to differentiate. The cells were grown at 37 °C in a 5 % CO_2_/95 % air humidified atmosphere. Cells were seeded on poly-L-lysine-coated plates and passaged at 70 %–80 % confluence. Experiments were carried out 24 h after the SH-SY5Y cells were seeded onto plates or dishes at an appropriate density. Cells were treated with phenolic amides (1-5) (40 μM) or different concentrations of LyA (10, 20, 40 μM) for 8 h before oxygen and glucose deprivation (OGD). To simulate ischemia-like conditions in vitro, cells were exposed to instantaneous OGD for 60 min. Then the cells were incubated again in the incubator with 5 % CO_2_ / 95 % air to terminate the OGD and start reperfusion for 24 h.

### Cell viability assay

MTT assay was used to measure cell viability in OGD experiments. SH-SY5Y cells were seeded in 96-well plates (1×10^4^ cells/well) at 37 °C in a 5 % CO_2_ / 95 % air incubator in DMEM media overnight. After the cells are subjected to the indicated treatments, 0.5 mg/mL MTT was added to each well and the cells were incubated at 37 °C for 4 h. Then the medium was carefully removed, and cells were lysed by shaking in 150 μL of dimethylsulfoxide (DMSO) for 10 min. The absorbance was determined at 490 nm using a microplate reader (Bio-Rad, Hercules, CA, USA). Cell viability was calculated and averaged. The control group were treated in the same manner without OGD, and cell viability was expressed as a percentage of the untreated control.

The cytotoxicity of OGD and the protective effect of LyA were further evaluated by the release of lactate dehydrogenase (LDH) into the incubation medium. SH-SY5Y cells were seeded in 96-well plates (1×10^4^ cells/well) at 37 °C in a 5 % CO_2_ / 95 % air incubator in DMEM media overnight. After the cells are subjected to the indicated treatments, the supernatant was used to determine LDH activity, which was determined by using an assay kit according to the manufacturer’s instructions. LDH leakage was expressed as the percentage of the total cell LDH activity. The LDH release was quantified by measuring the absorbance at 450 nm using a microplate reader (Elx800 Bio-Tek, USA).

### Hoechst 33342 staining

Hoechst 33342 staining were used to investigate changes in the nuclear morphology of apoptotic cells. The SH-SY5Y cells were grown on glass coverslips and treated with the indicated treatments, then cells were fixed with 4% paraformaldehyde for 15 min. Subsequently, cells were washed three times with PBS and incubated with 10 μg/mL Hoechst 33342 for 20 min at room temperature in the dark. Finally, the cells were examined using a fluorescence microscope (Olympus FV500, Japan).

### Flow cytometric analysis for apoptosis

Apoptotic cells were quantified using fluorescein isothiocyanate-labeled Annexin V binding and propidium iodide (PI) uptake. Briefly, at the end of indicated described, SH-SY5Y cells were harvested by centrifugation, washed two times with ice-cold PBS, resuspended in a Ca^2+^-enriched binding buffer at a concentration of 1×10^6^ cells/mL, and incubated with 5 μl Annexin V-FITC (20 μg/ml) and 10 μl PI (50 μg/ml) for 15 min at room temperature in the dark. Quantitative analysis of the level of apoptosis was performed using a flow cytometer (BD Biosciences, Franklin, NJ, USA).

### Measurement of intracellular ROS

Intracellular ROS was assessed by using the dihydroethidium (DHE) fluorescent probe. Briefly, at the end of indicated described, SH-SY5Y cells were incubated with 5 μM DHE for 30 min at 37 °C, washed twice and collected by centrifugation, then resuspended in PBS. Finally, the fluorescence intensity was measured by flow cytometer (BD Biosciences, Franklin, NJ, USA). The measured fluorescence values were expressed as a percentage of the fluorescence in control cells.

### Transient transfection with siRNA

Differentiated SH-SY5Y cells were transiently transfected with small interfering RNA (siRNA) against PKCε, Nrf2 or HO-1 by lipofectamine according to the manufacturer’s protocol (Santa Cruz Biotechnology, Inc., USA). Transfection was performed using commercially available human PKCε, Nrf2, HO-1-specific siRNAs (Santa Cruz) and negative control siRNAs (Santa Cruz). The efficiency of RNA interference was evaluated by western blot. Cells were transfected with siRNA or non-targeted interference control siRNA for 48 h, and then treated with LyA for the indicated time. Lastly, cell samples were collected for western blot analysis, MTT assay, and intracellular ROS level measurement.

### Western blot analysis

After reperfusion, ischemic brain slices including both the ischemic area and the corresponding regions of the contralateral hemisphere were collected. The tissues in the cortex were homogenized in RAPI lysis buffer (Beyotime, China), centrifuged at 14,000 g for 30 min at 4 °C, and then the supernatant was collected as total protein. The cells were lysed in cell lysis buffer, centrifuged at 16,000 g for 20 min at 4 °C, and then the supernatant was collected as total protein. To prepare the cytoplasmic and nuclear proteins, the tissues and cells were lysed using a nuclear and cytoplasmic protein extraction kit (Beyotime, China) according to the manufacturer’s instructions, respectively. Proteins were mixed with sample buffer, and equal loaded and separated by 10% SDS-PAGE gels, then transferred to polyvinylidene difluoride (PVDF) membranes. After blocking with 5% non-fat dry milk Tris-buffered saline (TBS) containing 0.1% Tween 20 at room temperature for 1 h, the membranes were incubated with primary antibody (p-PKCε, PKCε, Nrf2, HO-1, Bcl-2, Bax, caspase-3, β-actin and histone H3) overnight at 4 °C. Following that, the membranes were washed three times in TBST buffer, then incubated with the anti-rabbit or anti-mouse IgG secondary antibody in TBST for 1 h at room temperature. Finally, after three times of washing, blotted proteins were visualized with the ECL-chemiluminescent kit (GE Healthcare, Life Sciences) using the western blotting detection system (ECL Plus, Amersham, Buckinghamshire, UK).

### Statistical analysis

Data are expressed as mean ± SD and were analyzed by SPSS 18.0 (SPSS Inc., Chicago, IL, USA). Except for neurological score, statistical significance was analyzed with one-way ANOVA analysis of variance followed by Tukey’s multiple comparison tests. Neurological deficit scores were expressed as the median ranges, which was analyzed by the Kruskal-Wallis test followed by the Mann-Whitney U test and Bonferroni post hoc correction. Values of *p* < 0.05 were considered to be statistically significant.
